# Algorithms for converting estimates of child malnutrition based on the NCHS reference into estimates based on the WHO Child Growth Standards

**DOI:** 10.1186/1471-2431-8-19

**Published:** 2008-05-05

**Authors:** Hong Yang, Mercedes de Onis

**Affiliations:** 1 Department of Nutrition, World Health Organization, Geneva, Switzerland

## Abstract

**Background:**

The child growth standards released by the World Health Organization (WHO) in 2006 have several technical advantages over the previous 1977 National Center for Health Statistics (NCHS)/WHO reference and are recommended for international comparisons and secular trend analysis of child malnutrition. To obtain comparable data over time, earlier surveys should be reanalyzed using the WHO standards; however, reanalysis is impossible for older surveys since the raw data are not available. This paper provides algorithms for converting estimates of child malnutrition based on the NCHS reference into estimates based on the WHO standards.

**Methods:**

Sixty-eight surveys from the WHO Global Database on Child Growth and Malnutrition were analyzed using the WHO standards to derive estimates of underweight, stunting, wasting and overweight. The prevalences based on the NCHS reference were taken directly from the database. National/regional estimates with a minimum sample size of 400 children were used to develop the algorithms. For each indicator, a simple linear regression model was fitted, using the logit of WHO and NCHS estimates as, respectively, dependent and independent variables. The resulting algorithms were validated using a different set of surveys, on the basis of which the point estimate and 95% confidence interval (CI) of the predicted WHO prevalence were compared to the observed prevalence.

**Results:**

In total, 271 data points were used to develop the algorithms. The correlation coefficients (R^2^) were all greater than 0.90, indicating that most of the variability of the dependent variable is explained by the fitted model. The average difference between the predicted WHO estimate and the observed value was <0.5% for stunting, wasting and overweight. For underweight, the mean difference was 0.8%. The proportion of the 95% CI of the predicted estimate containing the observed prevalence was above 90% for all four indicators. The algorithms performed equally well for surveys without the entire age coverage 0 to 60 months.

**Conclusion:**

To obtain comparable data concerning child malnutrition, individual survey data should be analyzed using the WHO standards. When the raw data are not available, the algorithms presented here provide a highly accurate tool for converting existing NCHS estimates into WHO estimates.

## Background

In April 2006 the World Health Organization (WHO) released new standards for assessing the growth and development of children from birth to five years of age [1-3]. The WHO Child Growth Standards (hereafter referred to as the WHO standards) were developed to replace the National Center for Health Statistics (NCHS)/WHO international growth reference [4] (hereafter referred to as the NCHS reference), whose limitations have been described in detail elsewhere [5]. The new standards are based on an international sample of healthy children living under conditions likely to favour achievement of their full genetic growth potential. Furthermore, the mothers of the children selected for the construction of the standards engaged in fundamental health-promoting practices, namely breastfeeding and not smoking.

As anticipated, the substantially different approaches used to construct the NCHS reference and the WHO standards resulted in significant differences between the two. These differences vary by anthropometric measure, sex, specific percentile or z-score curve, age, and population-specific anthropometric characteristics [1,6]. The impact on population estimates of child malnutrition will therefore vary depending on all these features.

A notable effect is that stunting will be greater throughout childhood when assessed using the WHO standards compared to the NCHS reference. For underweight, the WHO standards will result in a substantial increase in rates of low weight-for-age during the first half of infancy (i.e., 0–6 months) and a decrease thereafter. For wasting, the main difference between the WHO standards and the NCHS reference is during infancy (i.e., up to about 70 cm in length) when wasting rates will be substantially higher using the WHO standards. With respect to overweight, use of the WHO standards will result in a greater prevalence that will vary according to the age, sex and nutritional status of the index population. A detailed description of the differences in the rates of underweight, stunting, wasting and overweight has been published elsewhere [6].

At present, the NCHS reference is used in the national nutrition surveillance programmes of over 100 countries [7]. Similarly, the United Nations system monitors the progress of nations in achieving the Millennium Development Goal of halving, between 1990 and 2015, the proportion of people who suffer from hunger, using as a basis for comparison the NCHS reference [8]. It is thus important to have comparable trend data on child malnutrition both for national and international use, and this will require the reanalysis of the earlier anthropometric surveys using the WHO standards. However, the analysis of the earlier surveys will not always be possible primarily due to lack of availability of the raw data. This is mainly the case for surveys conducted prior to the 1980s, for which data are not available in electronic form. As an alternative approach to producing comparable trend nutritional data, we developed algorithms that convert the estimates of child malnutrition based on the NCHS reference into estimates based on the WHO standards. This paper provides algorithms for the indicators of underweight, stunting, wasting and overweight, and evaluates their performance in predicting WHO-based estimates.

## Methods

To develop the algorithms, we selected from the WHO Global Database on Child Growth and Malnutrition [9] nutritional surveys for which raw data were available for analysis using the WHO standards. Where feasible in a given national survey, regional estimates were used in place of national ones in order to increase the number of data points for developing the algorithms. To ensure robustness, only estimates based on a minimum sample size of 400 children were included.

For each survey we generated prevalence estimates of underweight (percent below -2 standard deviation (SD) weight-for-age), stunting (percent below -2 SD length/height-for-age), wasting (percent below -2 SD weight-for-length/height), and overweight (percent above +2 SD weight-for-length/height) based on the WHO standards (WHO estimates). The prevalence estimates for the NCHS (NCHS estimates) were taken directly from the global database.

A simple linear regression was performed, using the NCHS and WHO prevalence estimates as, respectively, the independent and dependent variables. Since the prevalence scale is between 0 and 1, to perform the regression in the entire real line, the logit transformation was applied to both estimates before fitting them to the regression model.

Subsequently, the resulting algorithms (i.e., the simple linear regression models) were validated using a new set of surveys from the WHO global database that had not been included in the development of the algorithms. The predicted WHO estimate and its 95% confidence interval (95% CI) were estimated from the regression model. Since the regression was performed in the logit scale, its reverse transformation was applied to obtain the estimated values in the prevalence scale. The predicted WHO estimate was compared to the observed prevalence to examine the actual difference, predicted vs. observed; and the 95% CI of the predicted estimate was evaluated for its coverage of the observed. All analyses were performed using SAS version 8.2.

## Results

In total, 64 national and 4 research surveys were selected from the WHO global database. The age coverage of the surveys was: 0–60 months for 64 surveys, 0–36 months for 2 surveys, and 1 each for 3–60 and 6–60 months. The total number of national/regional estimates used for creating the algorithms for underweight, stunting and wasting was 271. For overweight, the sample size was decreased to 255 since the NCHS-based estimate of overweight was not available from the database for 16 surveys.

**Figure 1:**
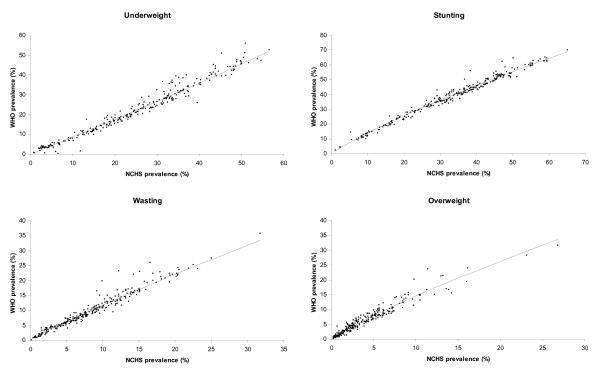
**Observed WHO and NCHS prevalence estimates and fitted regression lines in the algorithm estimation**. ▲: scatter plot of the observed WHO vs. NCHS prevalence estimates. --: fitted regression line in the prevalence scale.

The scatter plots of the two prevalence estimates, WHO vs. NCHS, used for algorithm estimation are presented in Figure 1. The observed estimates are very close to the fitted regression lines in the prevalence scale.

Table 1 presents the parameter estimates from the simple linear regressions in the logit scale. The correlation coefficients, R^2^, are all greater than 0.90, indicating that most of the variability of the dependent variable, logit (P_WHO_), can be explained by the fitted model for each algorithm. The point estimates of the slopes are greater than 0.9 for all four indicators; and assuming they are equal to "1", the odds ratios of the two prevalence estimates, WHO vs. NCHS, can be approximated by the exponential function of the intercept, i.e., exp [intercept], which are 0.84, 1.24, 1.03 and 1.26 for underweight, stunting, wasting and overweight, respectively. As shown in Table 1, among all the parameter estimates of the model, only the intercept from the wasting algorithm is not significant; the others, intercepts and slopes, are all significant at the 5% level, with their 95% CIs not including "0".

**Table 1:** Parameter estimates from the simple regression models

		Simple linear regression model in the logit^1 ^scale: logit (P_WHO_) = a+b*logit (P_NCHS_)			
		
Algorithm	N	Intercept a (95% CI)	Slope b (95% CI)	Correlation coefficient (R^2^)	Mean square error
Underweight	271	-0.177 (-0.231, -0.124)^2^	0.987 (0.955, 1.019)^2^	0.931	0.073
Stunting	271	0.216 (0.198, 0.235)^2^	0.925 (0.908, 0.941)^2^	0.979	0.012
Wasting	271	0.026 (-0.038, 0.090)	0.928 (0.905, 0.951)^2^	0.959	0.030
Overweight	256	0.235 (0.117, 0.353)^2^	0.912 (0.880, 0.944)^2^	0.925	0.064

^1 ^logit (p) = log [P/(1-P)], where P is either PWHO or PNCHS, the WHO and NCHS prevalence estimates, respectively.

^2 ^p-value < 0.05

To validate the performance of the algorithms, 65 national/regional surveys that were not included in their estimation were selected from the WHO global database. Of these, 16 surveys did not cover the entire 0–60 months age range. For overweight, NCHS-based prevalence estimates were available for 56 surveys only. Additional file 1 presents, for the four anthropometric indicators, the observed WHO and NCHS prevalence estimates from the 65 surveys, the predicted WHO estimates with the 95% CI, and the actual difference between the predicted and the observed WHO prevalences.

**Figure 2:**
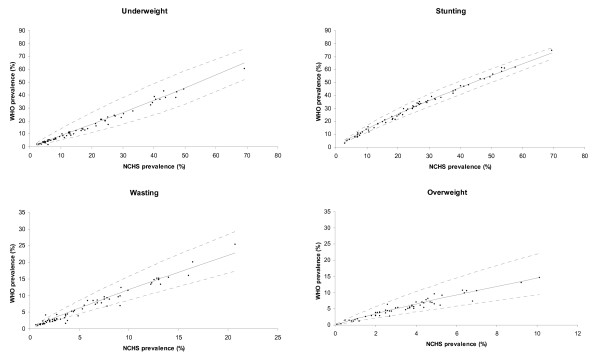
**NCHS and WHO observed estimates and the predicted WHO estimates from the algorithm validation**. ▲: scatter plot of the observed WHO vs. NCHS prevalence estimates. --: line connecting the predicted WHO estimates. ---: line connecting the 95% CI limits (lower or upper) of the predicted WHO estimates.

The scatter plots of the two prevalence estimates, WHO vs. NCHS, used for the algorithm validation are presented along with the predicted lines and their 95% CI bands in Figure 2. Since the linear regression models were fitted in the logit scale, the 95% CI bands in the prevalence scale have funnel shapes. Almost all the observed WHO estimates are within the 95% CI bands.

The differences between the predicted and the observed WHO estimates were examined, and their summary statistics are presented in Table 2. The average difference is less than a half percent (0.5%) for stunting, wasting and overweight. For underweight, the mean difference is slightly higher (0.8%). In the lower prevalence group, i.e., <5%, the magnitude of the difference remains roughly the same except for stunting, where the mean difference is a bit higher (0.7%) due to the small number of surveys in this category (n = 3).

**Table 2:** Summary results from the algorithm validation

Algorithm	Prevalence	N	Mean difference Predicted minus observed WHO prevalence (SD)	Percentage of the observed WHO prevalence within the 95% CI of the predicted values (%)
Underweight	<5%	9	0.2 (0.46)	100
	≥5%	56	0.9 (1.62)	98.2
	Total	65	0.8 (1.53)	98.5
Stunting	<5%	3	0.7 (0.55)	66.7
	≥5%	62	0.1 (1.34)	96.8
	Total	65	0.1 (1.32)	95.4
Wasting	<5%	36	0.4 (0.86)	88.9
	≥5%	29	0.2 (1.30)	96.6
	Total	65	0.3 (1.07)	92.3
Overweight	<5%	46	0.2 (0.74)	97.8
	≥5%	9	0.3 (1.36)	100
	Total	55	0.2 (0.85)	98.2

The percentage of the 95% CI of the predicted estimate containing the observed prevalence is above 95% for underweight, stunting and overweight, whereas it is slightly lower (92.3%) for wasting (Table 2). This magnitude of coverage remains roughly the same in the lower prevalence category.

Finally, the four algorithms for calculating the predicted WHO estimates with 95% CI are presented in an Excel programme in Additional file 2. An NCHS prevalence estimate in percentage is expected, from which the programme will generate the WHO-based point estimates with 95% CIs for the four indicators.

## Discussion

This paper provides algorithms that convert estimates of child malnutrition based on the NCHS reference into estimates based on the WHO Child Growth Standards. The algorithms were developed using simple linear regression models fitted in the logit scale. Empirically, the linear assumption between the NCHS and WHO estimates in the logit scale was supported by the observed relationship displayed in their scatter plots along with the fitted lines in the prevalence scale (Figure 1). The linear regression model has been used in the past to estimate rates of intrauterine growth retardation [10], and its robustness and high predictability have been demonstrated by de Onis et al [11].

In estimating the algorithms, national/regional estimates were taken from nutritional surveys included in the WHO global database for which WHO estimates were derived using raw data. Potential outliers were examined; however, since there was no evidence of data quality errors, they were not excluded in fitting the models. This approach should preserve the robustness of the models, as a reflection of real life, while keeping the sample consistent in estimating the four algorithms.

In all four algorithms, the estimated slopes are close to "1", and except for the underweight algorithm, the estimated intercepts are large than "0"; this indicates that, in general, the predicted WHO estimates are higher than the respective NCHS estimates for stunting, wasting and overweight, but that the reverse applies for underweight. As shown in an earlier paper from our group [6] with regard to weight-for-age, the average weight of infants included in the WHO standards is above the NCHS median during the first half of infancy, crosses it at about 6 months, and tracks below it thereafter. Therefore, the pattern of the difference between the NCHS reference and the WHO standards estimates depends on the specific age interval. The prevalence of underweight during the first six months of life will be higher when using the WHO standards compared to the NCHS reference, but lower thereafter [6]. For a survey covering the entire age group, i.e., 0–60 months, the age-combined prevalence (a weighted average) for underweight is strongly influenced by the majority of late age, and the result is a lower WHO estimate. This is clearly demonstrated in its algorithm, with an approximate OR_WHO/NCHS _= 0.84. For wasting, there is also a crossing of centiles at about 72 cm of length (equivalent to 9 months in a healthy population or around 1 year in a stunted population), but thereafter the -2 SD lines for the NCHS and the WHO curves largely overlap [1]. Therefore, although in general the prevalence of wasting for the entire range 0–60 months is somewhat higher when based on the WHO standards compared to the NCHS reference, occasionally it might be lower depending on the age-specific length/height distribution of the index population. This is reflected in the wasting algorithm, which usually yields a slightly higher WHO-predicted estimate (see Additional file 1) with an approximated OR_WHO/NCHS _= 1.03.

The four algorithms have a high degree of predictability as demonstrated by the validation results (Additional file 1). On average, the magnitude of the differences is very small, less than 0.5% for stunting, wasting and overweight (Table 2). For stunting, this difference is almost negligible given that stunting rates are above 20% in most developing countries. Also, in almost all cases the observed WHO prevalence falls within the 95% CIs of the predicted estimate (Table 2), especially for underweight, stunting and overweight, where more than 95% of the observed WHO estimates are within the confidence limits. Similarly, the small magnitude of the mean differences and high coverage of the 95% CIs are affected only slightly, if at all, when the prevalence is low (i.e., below 5%)(Table 2).

The majority of the surveys used in developing the algorithms cover the age range 0 to 60 months. Nevertheless, the algorithms can also be applied to convert NCHS estimates from surveys with a different age range (e.g., 6–60 months and 3–36 months) since the predicted WHO estimates are very close to the observed values (Additional file 1). Although in some cases the difference increases slightly when the survey covers an age range under 0–60 months, most of the 95% CIs still cover the observed WHO estimate.

## Conclusion

In summary, the WHO standards have several technical advantages over the NCHS reference, including its source population, study design and statistical methods applied to construct the curves [2], and WHO and other international bodies such as the International Pediatric Association [12] recommend them for use with individual children and in child populations. To obtain comparable data for international comparisons and for secular trend analysis, the WHO estimates should be derived using raw data whenever possible. However, for those surveys for which raw data are not available, the algorithms presented here provide an easy-to-use tool for calculating accurately the WHO estimates using the historical NCHS-based estimates.

## Competing interests

The authors declare that they have no competing interests.

## Authors' contributions

HY and MdO developed the idea for the analysis. HY performed the analysis and drafted the manuscript. MdO guided the analysis and interpretation of results and revised the manuscript for essential intellectual content.

## Pre-publication history

The pre-publication history for this paper can be accessed here:



## Supplementary Material

Additional file 1 This file contains a table presenting the results of the validation described in the results section of the manuscript. It is under the title "Individual NCHS and WHO observed estimates and the predicted WHO estimates from the algorithm validation". It is a standard word file created by MS Word 2003. Click here for file

Additional file 2 This Excel programme contains the actual algorithms developed in this paper. For each indicator, i.e. underweight, stunting, wasting and overweight, a NCHS-based prevalence estimate in percentage is expected from the user, from which the predicted WHO-based estimate with its 95% CI will be generated by the programme. Click here for file

## References

[B1] WHO Multicentre Growth Reference Study Group (2006). WHO Child Growth Standards: Length/height-for-age, weight-for-age, weight-for-length, weight-for-height and body mass index-for-age: Methods and development.

[B2] WHO Multicentre Growth Reference Study Group (2006). WHO Child Growth Standards based on length/height, weight and age. Acta Paediatr Suppl.

[B3] Garza C, de Onis M, for the WHO Multicentre Growth Reference Study Group (2004). Rationale for developing a new international growth reference. Food Nutr Bull.

[B4] Hamill PVV, Drizd TA, Johnson CL, Reed RB, Roche AF, Moore WM (1979). Physical growth: National Center for Health Statistics percentiles. Am J Clin Nutr.

[B5] de Onis M, Yip R (1996). The WHO growth chart: historical considerations and current scientific issues. Bibl Nutr Dieta.

[B6] de Onis M, Onyango AW, Borghi E, Garza C, Yang H, for the WHO Multicentre Growth Reference Study Group (2006). Comparison of the WHO Child Growth Standards and the NCHS/WHO international growth reference: implications for child health programmes. Public Health Nutr.

[B7] de Onis M, Wijnhoven TMA, Onyango AW (2004). Worldwide practices in child growth monitoring. J Pediatr.

[B8] UN Millennium Project 2005 (2005). Halving Hunger: It Can Be Done Task Force on Hunger 2005.

[B9] de Onis M, Bloessner M (2003). The World Health Organization Global Database on Child Growth and Malnutrition: methodology and applications. Int J Epidemiol.

[B10] Villar J, Ezcurra EJ, Gurtner de la Fuente V, Campodonico L (1994). Pre-term delivery syndrome: the unmet need. New perspectives for the effective treatment of pre-term labor: an international consensus Research and Clinical Forums.

[B11] de Onis M, Bloessner M, Villar J (1998). Levels and patterns of intrauterine growth retardation in developing countries. Eur J Clin Nutr.

[B12] International Pediatric Association. Endorsement of the New WHO Growth Standards for Infants and Young Children. http://www.who.int/childgrowth/Endorsement_IPA.pdf.

