# Supplementary material for: Algorithms for converting estimates of child malnutrition based on the NCHS reference into estimates based on the WHO Child Growth Standards
Source: BMC Pediatr. 2008 May 5;8:19. doi: 10.1186/1471-2431-8-19 (PMC2390546; doi:10.1186/1471-2431-8-19)
Supplement: Additional file 1 — This file contains a table presenting the results of the validation described in the results section of the manuscript. It is under the title "Individual NCHS and WHO observed estimates and the predicted WHO estimates from the algorithm validation". It is a standard word file created by MS Word 2003. [file 1471-2431-8-19-S1.doc]

Individual NCHS and WHO observed estimates and the predicted WHO estimates from the algorithm validation

|  |  |  |  |  | Observed prevalence (%) | | Predicted WHO prevalence (%) | |
| --- | --- | --- | --- | --- | --- | --- | --- | --- |
| Country | Survey year | Age | N | Prevalence | NCHS-based | WHO-based | Point estimate (95 %CI) | Actual difference  predicted minus observed |
|  |  |  |  |  |  |  |  |  |
| Algeria | 1995 | 0-60 mo | 3825 | Underweight | 12.8 | 11.3 | 11.2 (6.9 , 17.7) | -0.1 |
|  |  |  |  | Stunting | 18.3 | 22.5 | 23.7 (20.1 , 27.8) | 1.2 |
|  |  |  |  | Wasting | 8.9 | 9.6 | 10.6 (7.8 , 14.3) | 1 |
|  |  |  |  | Overweight | 9.2 | 13.2 | 13.5 (8.7 , 20.6) | 0.3 |
|  |  |  |  |  |  |  |  |  |
|  | 2000 | 0-60 mo | 4178 | Underweight | 6 | 5.4 | 5.3 (3.1 , 8.6) | -0.1 |
|  |  |  |  | Stunting | 18 | 23.6 | 23.4 (19.8 , 27.4) | -0.2 |
|  |  |  |  | Wasting | 2.7 | 3.1 | 3.6 (2.6 , 4.9) | 0.5 |
|  |  |  |  | Overweight | 10.1 | 14.7 | 14.7 (9.4 , 22.1) | 0 |
|  |  |  |  |  |  |  |  |  |
|  | 2002 | 0-60 mo | 2610 | Underweight | 10.4 | 10.2 | 9.1 (5.5 , 14.6) | -1.1 |
|  |  |  |  | Stunting | 19.1 | 21.6 | 24.6 (20.9 , 28.8) | 3 |
|  |  |  |  | Wasting | 7.5 | 9.7 | 9.1 (6.6 , 12.3) | -0.6 |
|  |  |  |  |  |  |  |  |  |
| Angola | 1996 | 6-60 mo | 3016 | Underweight | 40.6 | 37 | 36.5 (25.2 , 49.5) | -0.5 |
|  |  |  |  | Stunting | 53.3 | 61.7 | 58.4 (53.1 , 63.4) | -3.3 |
|  |  |  |  | Wasting | 5.8 | 8.6 | 7.2 (5.2 , 9.8) | -1.4 |
|  |  |  |  | Overweight | 0.5 | 1.6 | 1 (0.6 , 1.6) | -0.6 |
|  |  |  |  |  |  |  |  |  |
| Armenia | 1998 | 0-60 mo | 3241 | Underweight | 3.3 | 2.7 | 2.9 (1.7 , 4.9) | 0.2 |
|  |  |  |  | Stunting | 12.3 | 15.1 | 16.8 (14 , 20) | 1.7 |
|  |  |  |  | Wasting | 3.5 | 3.3 | 4.5 (3.3 , 6.2) | 1.2 |
|  |  |  |  | Overweight | 6.3 | 10.8 | 9.7 (6.1 , 15.1) | -1.1 |
|  |  |  |  |  |  |  |  |  |
| Azerbaijan | 2000 | 0-60 mo | 1789 | Underweight | 16.8 | 14 | 14.7 (9.2 , 22.7) | 0.7 |
|  |  |  |  | Stunting | 19.6 | 24.1 | 25.2 (21.4 , 29.4) | 1.1 |
|  |  |  |  | Wasting | 8 | 9 | 9.6 (7 , 13) | 0.6 |
|  |  |  |  | Overweight | 3.8 | 6.2 | 6.2 (3.9 , 9.9) | 0 |
|  |  |  |  |  |  |  |  |  |
| Barbados | 1981 | 0-60 mo | 620 | Underweight | 5.9 | 5.3 | 5.2 (3.1 , 8.5) | -0.1 |
|  |  |  |  | Stunting | 7 | 10.2 | 10.2 (8.4 , 12.3) | 0 |
|  |  |  |  | Wasting | 4.9 | 3.9 | 6.1 (4.5 , 8.4) | 2.2 |
|  |  |  |  | Overweight | 3.9 | 5.7 | 6.4 (4 , 10.1) | 0.7 |
|  |  |  |  |  |  |  |  |  |
| Benin | 2001 | 0-60 mo | 4947 | Underweight | 22.9 | 21.5 | 20.2 (12.9 , 30.1) | -1.3 |
|  |  |  |  | Stunting | 30.7 | 39.1 | 36.9 (32.1 , 42) | -2.2 |
|  |  |  |  | Wasting | 7.5 | 9 | 9.1 (6.6 , 12.3) | 0.1 |
|  |  |  |  | Overweight | 1.8 | 3 | 3.2 (2 , 5.2) | 0.2 |
|  |  |  |  |  |  |  |  |  |
| Bhutan | 1999 | 6-60 mo | 2996 | Underweight | 18.7 | 14.1 | 16.4 (10.3 , 25.1) | 2.3 |
|  |  |  |  | Stunting | 40 | 47.7 | 46 (40.8 , 51.3) | -1.7 |
|  |  |  |  | Wasting | 2.6 | 2.5 | 3.4 (2.5 , 4.8) | 0.9 |
|  |  |  |  | Overweight | 2.1 | 3.9 | 3.7 (2.3 , 5.9) | -0.2 |
|  |  |  |  |  |  |  |  |  |
| Bolivia | 1989 | 3-36 mo | 2563 | Underweight | 13.2 | 8.9 | 11.5 (7.1 , 18.2) | 2.6 |
|  |  |  |  | Stunting | 37.7 | 41.7 | 43.8 (38.7 , 49.1) | 2.1 |
|  |  |  |  | Wasting | 1.6 | 2.2 | 2.2 (1.6 , 3.1) | 0 |
|  |  |  |  | Overweight | 4.5 | 7.8 | 7.2 (4.5 , 11.4) | -0.6 |
|  |  |  |  |  |  |  |  |  |
|  | 1993-94 | 3-36 mo | 2730 | Underweight | 14.9 | 12.6 | 13 (8.1 , 20.4) | 0.4 |
|  |  |  |  | Stunting | 26.8 | 35.2 | 32.9 (28.4 , 37.7) | -2.3 |
|  |  |  |  | Wasting | 4.2 | 5.3 | 5.3 (3.9 , 7.3) | 0 |
|  |  |  |  | Overweight | 4.3 | 7.1 | 6.9 (4.3 , 11) | -0.2 |
|  |  |  |  |  |  |  |  |  |
|  | 1998 | 0-60 mo | 6005 | Underweight | 7.6 | 5.9 | 6.6 (4 , 10.8) | 0.7 |
|  |  |  |  | Stunting | 26.8 | 33.1 | 32.9 (28.4 , 37.7) | -0.2 |
|  |  |  |  | Wasting | 1.3 | 1.6 | 1.8 (1.3 , 2.5) | 0.2 |
|  |  |  |  | Overweight | 6.5 | 10.7 | 10 (6.3 , 15.5) | -0.7 |
|  |  |  |  |  |  |  |  |  |
| Brazil | 1996 | 0-60 mo | 4145 | Underweight | 5.7 | 4.5 | 5 (3 , 8.2) | 0.5 |
|  |  |  |  | Stunting | 10.5 | 13.5 | 14.6 (12.1 , 17.5) | 1.1 |
|  |  |  |  | Wasting | 2.3 | 2.8 | 3.1 (2.2 , 4.3) | 0.3 |
|  |  |  |  | Overweight | 4.9 | 6.6 | 7.8 (4.9 , 12.2) | 1.2 |
|  |  |  |  |  |  |  |  |  |
| Cambodia | 1996 | 0-60 mo | 5773 | Underweight | 47.4 | 42.6 | 43 (30.7 , 56.3) | 0.4 |
|  |  |  |  | Stunting | 53.3 | 58.6 | 58.4 (53.1 , 63.4) | -0.2 |
|  |  |  |  | Wasting | 12.2 | 13.4 | 14.1 (10.5 , 18.8) | 0.7 |
|  |  |  |  | Overweight | 2.6 | 6.5 | 4.4 (2.7 , 7.1) | -2.1 |
|  |  |  |  |  |  |  |  |  |
| Colombia | 1995 | 0-60 mo | 4458 | Underweight | 8.4 | 6.3 | 7.3 (4.4 , 11.9) | 1 |
|  |  |  |  | Stunting | 15 | 19.7 | 20 (16.8 , 23.6) | 0.3 |
|  |  |  |  | Wasting | 1.4 | 1.7 | 1.9 (1.4 , 2.7) | 0.2 |
|  |  |  |  | Overweight | 2.6 | 4.5 | 4.4 (2.7 , 7.1) | -0.1 |
|  |  |  |  |  |  |  |  |  |
|  | 2000 | 0-60 mo | 4119 | Underweight | 6.7 | 4.9 | 5.9 (3.5 , 9.6) | 1 |
|  |  |  |  | Stunting | 13.5 | 18.1 | 18.2 (15.3 , 21.6) | 0.1 |
|  |  |  |  | Wasting | 0.8 | 1.1 | 1.2 (0.8 , 1.6) | 0.1 |
|  |  |  |  | Overweight | 3.7 | 5.3 | 6.1 (3.8 , 9.6) | 0.8 |
|  |  |  |  |  |  |  |  |  |
| Cote D'Ivoire | 1998-99 | 0-60 mo | 1772 | Underweight | 21.2 | 18.2 | 18.6 (11.9 , 28.1) | 0.4 |
|  |  |  |  | Stunting | 25.1 | 31.5 | 31.1 (26.8 , 35.8) | -0.4 |
|  |  |  |  | Wasting | 7.8 | 6.9 | 9.4 (6.9 , 12.7) | 2.5 |
|  |  |  |  | Overweight | 2.5 | 4.6 | 4.3 (2.6 , 6.9) | -0.3 |
|  |  |  |  |  |  |  |  |  |
| Dominican Republic | 2000 | 0-60 mo | 1907 | Underweight | 4.6 | 3.5 | 4 (2.4 , 6.7) | 0.5 |
|  |  |  |  | Stunting | 6.1 | 8 | 9 (7.4 , 10.9) | 1 |
|  |  |  |  | Wasting | 1.5 | 1.5 | 2.1 (1.5 , 2.9) | 0.6 |
|  |  |  |  |  |  |  |  |  |
| El Salvador | 1993 | 3-60 mo | 3598 | Underweight | 11.2 | 7.2 | 9.8 (6 , 15.6) | 2.6 |
|  |  |  |  | Stunting | 23.1 | 29.5 | 29 (24.8 , 33.5) | -0.5 |
|  |  |  |  | Wasting | 1.3 | 1.4 | 1.8 (1.3 , 2.5) | 0.4 |
|  |  |  |  | Overweight | 2.2 | 3.9 | 3.8 (2.3 , 6.1) | -0.1 |
|  |  |  |  |  |  |  |  |  |
| Eritrea | 1995-96 | 3-36 mo | 2371 | Underweight | 43.7 | 38.3 | 39.5 (27.7 , 52.7) | 1.2 |
|  |  |  |  | Stunting | 38.4 | 44.4 | 44.5 (39.3 , 49.8) | 0.1 |
|  |  |  |  | Wasting | 16.4 | 20.2 | 18.5 (13.9 , 24.1) | -1.7 |
|  |  |  |  | Overweight | 0.9 | 1.2 | 1.7 (1 , 2.8) | 0.5 |
|  |  |  |  |  |  |  |  |  |
|  | 2002 | 0-60 mo | 5707 | Underweight | 39.6 | 34.5 | 35.6 (24.5 , 48.5) | 1.1 |
|  |  |  |  | Stunting | 37.6 | 43.7 | 43.7 (38.6 , 49) | 0 |
|  |  |  |  | Wasting | 12.5 | 14.9 | 14.4 (10.7 , 19.2) | -0.5 |
|  |  |  |  | Overweight | 0.7 | 1.6 | 1.4 (0.8 , 2.2) | -0.2 |
|  |  |  |  |  |  |  |  |  |
| Gabon | 2000-01 | 0-60 mo | 3204 | Underweight | 11.9 | 8.8 | 10.4 (6.4 , 16.5) | 1.6 |
|  |  |  |  | Stunting | 20.7 | 26.3 | 26.4 (22.5 , 30.7) | 0.1 |
|  |  |  |  | Wasting | 2.7 | 4.3 | 3.6 (2.6 , 4.9) | -0.7 |
|  |  |  |  | Overweight | 3.7 | 5.6 | 6.1 (3.8 , 9.6) | 0.5 |
|  |  |  |  |  |  |  |  |  |
| Georgia | 2000 | 0-60 mo | 3938 | Underweight | 2.8 | 2.2 | 2.5 (1.5 , 4.1) | 0.3 |
|  |  |  |  | Stunting | 8 | 10.9 | 11.5 (9.5 , 13.8) | 0.6 |
|  |  |  |  | Wasting | 0.6 | 1.2 | 0.9 (0.6 , 1.2) | -0.3 |
|  |  |  |  | Overweight | 4.6 | 8.3 | 7.4 (4.6 , 11.6) | -0.9 |
|  |  |  |  |  |  |  |  |  |
| Guatemala | 1987 | 3-36 mo | 2227 | Underweight | 33.2 | 27.8 | 29.6 (19.8 , 41.7) | 1.8 |
|  |  |  |  | Stunting | 57.7 | 62.1 | 62.3 (57.2 , 67.2) | 0.2 |
|  |  |  |  | Wasting | 1.3 | 2.4 | 1.8 (1.3 , 2.5) | -0.6 |
|  |  |  |  | Overweight | 2.7 | 2.8 | 4.6 (2.8 , 7.3) | 1.8 |
|  |  |  |  |  |  |  |  |  |
|  | 1998-99 | 0-60 mo | 3762 | Underweight | 24.2 | 20.3 | 21.3 (13.7 , 31.6) | 1 |
|  |  |  |  | Stunting | 46.4 | 53.1 | 52.1 (46.8 , 57.3) | -1 |
|  |  |  |  | Wasting | 2.5 | 2.9 | 3.3 (2.4 , 4.6) | 0.4 |
|  |  |  |  | Overweight | 4.4 | 6.9 | 7.1 (4.4 , 11.2) | 0.2 |
|  |  |  |  |  |  |  |  |  |
| Guinea | 1999 | 0-60 mo | 4615 | Underweight | 23.2 | 21.2 | 20.4 (13.1 , 30.5) | -0.8 |
|  |  |  |  | Stunting | 26.1 | 34.3 | 32.2 (27.7 , 37) | -2.1 |
|  |  |  |  | Wasting | 9.1 | 9.9 | 10.8 (8 , 14.6) | 0.9 |
|  |  |  |  | Overweight | 2.7 | 4.3 | 4.6 (2.8 , 7.3) | 0.3 |
|  |  |  |  |  |  |  |  |  |
| Haiti | 1994-95 | 0-60 mo | 2929 | Underweight | 27.5 | 24 | 24.3 (15.9 , 35.4) | 0.3 |
|  |  |  |  | Stunting | 31.9 | 37.2 | 38.1 (33.2 , 43.2) | 0.9 |
|  |  |  |  | Wasting | 7.8 | 9.4 | 9.4 (6.9 , 12.7) | 0 |
|  |  |  |  | Overweight | 2.8 | 4.3 | 4.7 (2.9 , 7.6) | 0.4 |
|  |  |  |  |  |  |  |  |  |
|  | 2000 | 0-60 mo | 6293 | Underweight | 17.2 | 13.9 | 15.1 (9.4 , 23.2) | 1.2 |
|  |  |  |  | Stunting | 22.7 | 28.3 | 28.6 (24.4 , 33.1) | 0.3 |
|  |  |  |  | Wasting | 4.5 | 5.6 | 5.7 (4.1 , 7.8) | 0.1 |
|  |  |  |  | Overweight | 2 | 3.1 | 3.5 (2.2 , 5.7) | 0.4 |
|  |  |  |  |  |  |  |  |  |
| Honduras | 2001 | 3-60 mo | 5643 | Underweight | 16.6 | 12.5 | 14.5 (9.1 , 22.5) | 2 |
|  |  |  |  | Stunting | 29.2 | 34.5 | 35.4 (30.7 , 40.4) | 0.9 |
|  |  |  |  | Wasting | 1.1 | 1.2 | 1.6 (1.1 , 2.2) | 0.4 |
|  |  |  |  | Overweight | 2.2 | 3 | 3.8 (2.3 , 6.1) | 0.8 |
|  |  |  |  |  |  |  |  |  |
| Hungary | 1980-88 | 0-60 mo | 70415 | Underweight | 2.2 | 2.3 | 1.9 (1.1 , 3.3) | -0.4 |
|  |  |  |  | Stunting | 2.9 | 3.3 | 4.6 (3.7 , 5.7) | 1.3 |
|  |  |  |  | Wasting | 1.6 | 4.2 | 2.2 (1.6 , 3.1) | -2 |
|  |  |  |  | Overweight | 2 | 3 | 3.5 (2.2 , 5.7) | 0.5 |
|  |  |  |  |  |  |  |  |  |
| Iran | 1980 | 0-60 mo | 774 | Underweight | 43.1 | 43.5 | 38.9 (27.2 , 52.1) | -4.6 |
|  |  |  |  | Stunting | 50.4 | 56.7 | 55.8 (50.5 , 60.9) | -0.9 |
|  |  |  |  | Wasting | 13 | 14.9 | 15 (11.1 , 19.8) | 0.1 |
|  |  |  |  | Overweight | 6.8 | 7.4 | 10.4 (6.6 , 16.1) | 3 |
|  |  |  |  |  |  |  |  |  |
| Jamaica | 1994 | 0-60 mo | 982 | Underweight | 4.6 | 4.5 | 4 (2.4 , 6.7) | -0.5 |
|  |  |  |  | Stunting | 10.7 | 11.9 | 14.9 (12.4 , 17.8) | 3 |
|  |  |  |  | Wasting | 3.1 | 2.9 | 4 (2.9 , 5.6) | 1.1 |
|  |  |  |  | Overweight | 5.2 | 6.2 | 8.2 (5.1 , 12.9) | 2 |
|  |  |  |  |  |  |  |  |  |
|  | 1995 | 0-60 mo | 959 | Underweight | 5.1 | 4 | 4.5 (2.7 , 7.4) | 0.5 |
|  |  |  |  | Stunting | 7.6 | 9.5 | 11 (9.1 , 13.2) | 1.5 |
|  |  |  |  | Wasting | 3.8 | 4.5 | 4.9 (3.5 , 6.7) | 0.4 |
|  |  |  |  | Overweight | 3.9 | 5.2 | 6.4 (4 , 10.1) | 1.2 |
|  |  |  |  |  |  |  |  |  |
|  | 1996 | 0-60 mo | 1002 | Underweight | 6.3 | 5.6 | 5.5 (3.3 , 9.1) | -0.1 |
|  |  |  |  | Stunting | 7 | 11.1 | 10.2 (8.4 , 12.3) | -0.9 |
|  |  |  |  | Wasting | 2.5 | 2.5 | 3.3 (2.4 , 4.6) | 0.8 |
|  |  |  |  | Overweight | 2.5 | 3.8 | 4.3 (2.6 , 6.9) | 0.5 |
|  |  |  |  |  |  |  |  |  |
|  | 1997 | 0-60 mo | 932 | Underweight | 4.2 | 3.5 | 3.7 (2.2 , 6.1) | 0.2 |
|  |  |  |  | Stunting | 6.9 | 8.6 | 10.1 (8.3 , 12.2) | 1.5 |
|  |  |  |  | Wasting | 2.3 | 2.3 | 3.1 (2.2 , 4.3) | 0.8 |
|  |  |  |  | Overweight | 4.4 | 5.5 | 7.1 (4.4 , 11.2) | 1.6 |
|  |  |  |  |  |  |  |  |  |
|  | 1998 | 0-60 mo | 3142 | Underweight | 5 | 3.4 | 4.4 (2.6 , 7.3) | 1 |
|  |  |  |  | Stunting | 6.2 | 8.3 | 9.1 (7.5 , 11.1) | 0.8 |
|  |  |  |  | Wasting | 2.7 | 2.9 | 3.6 (2.6 , 4.9) | 0.7 |
|  |  |  |  | Overweight | 3.6 | 5.2 | 5.9 (3.7 , 9.4) | 0.7 |
|  |  |  |  |  |  |  |  |  |
|  | 1999 | 0-60 mo | 810 | Underweight | 3.8 | 2.3 | 3.3 (2 , 5.6) | 1 |
|  |  |  |  | Stunting | 4.4 | 6.3 | 6.7 (5.5 , 8.2) | 0.4 |
|  |  |  |  | Wasting | 3.8 | 2.5 | 4.9 (3.5 , 6.7) | 2.4 |
|  |  |  |  | Overweight | 3.8 | 5.9 | 6.2 (3.9 , 9.9) | 0.3 |
|  |  |  |  |  |  |  |  |  |
| Jordan | 2002 | 0-60 mo | 4678 | Underweight | 4.4 | 3.6 | 3.9 (2.3 , 6.4) | 0.3 |
|  |  |  |  | Stunting | 8.5 | 12 | 12.1 (10 , 14.6) | 0.1 |
|  |  |  |  | Wasting | 2 | 2.5 | 2.7 (1.9 , 3.8) | 0.2 |
|  |  |  |  | Overweight | 3.5 | 4.7 | 5.8 (3.6 , 9.2) | 1.1 |
|  |  |  |  |  |  |  |  |  |
| Kazakhstan | 1999 | 0-60 mo | 626 | Underweight | 4.2 | 3.8 | 3.7 (2.2 , 6.1) | -0.1 |
|  |  |  |  | Stunting | 9.7 | 13.9 | 13.6 (11.3 , 16.3) | -0.3 |
|  |  |  |  | Wasting | 1.8 | 2.5 | 2.4 (1.8 , 3.4) | -0.1 |
|  |  |  |  | Overweight | 3 | 5.3 | 5 (3.1 , 8) | -0.3 |
|  |  |  |  |  |  |  |  |  |
| Madagascar | 2003-04 | 0-60 mo | 5905 | Underweight | 41.9 | 36.8 | 37.8 (26.2 , 50.9) | 1 |
|  |  |  |  | Stunting | 47.7 | 52.8 | 53.3 (48 , 58.5) | 0.5 |
|  |  |  |  | Wasting | 12.8 | 15.2 | 14.8 (11 , 19.6) | -0.4 |
|  |  |  |  |  |  |  |  |  |
| Maldives | 1994 | 0-60 mo | 1995 | Underweight | 39 | 32.5 | 35 (24 , 47.9) | 2.5 |
|  |  |  |  | Stunting | 29.6 | 36.1 | 35.8 (31.1 , 40.8) | -0.3 |
|  |  |  |  | Wasting | 16 | 16.1 | 18.1 (13.6 , 23.6) | 2 |
|  |  |  |  |  |  |  |  |  |
|  | 2001 | 0-60 mo | 746 | Underweight | 30.2 | 25.7 | 26.8 (17.7 , 38.4) | 1.1 |
|  |  |  |  | Stunting | 24.8 | 31.9 | 30.8 (26.5 , 35.5) | -1.1 |
|  |  |  |  | Wasting | 13.2 | 13.4 | 15.2 (11.3 , 20.1) | 1.8 |
|  |  |  |  | Overweight | 2 | 3.9 | 3.5 (2.2 , 5.7) | -0.4 |
|  |  |  |  |  |  |  |  |  |
| Mongolia | 1999 | 0-60 mo | 4146 | Underweight | 12.5 | 10.8 | 10.9 (6.7 , 17.3) | 0.1 |
|  |  |  |  | Stunting | 24.6 | 30.1 | 30.6 (26.3 , 35.3) | 0.5 |
|  |  |  |  | Wasting | 3.6 | 4.3 | 4.6 (3.3 , 6.4) | 0.3 |
|  |  |  |  | Overweight | 4.8 | 7 | 7.7 (4.8 , 12) | 0.7 |
|  |  |  |  |  |  |  |  |  |
|  | 2000 | 0-60 mo | 5918 | Underweight | 12.7 | 11.6 | 11.1 (6.8 , 17.6) | -0.5 |
|  |  |  |  | Stunting | 24.6 | 29.8 | 30.6 (26.3 , 35.3) | 0.8 |
|  |  |  |  | Wasting | 5.5 | 7.1 | 6.8 (5 , 9.3) | -0.3 |
|  |  |  |  |  |  |  |  |  |
| Nepal | 1975 | 6-60 mo | 5457 | Underweight | 69.1 | 60.8 | 64.9 (52 , 76) | 4.1 |
|  |  |  |  | Stunting | 69.4 | 75 | 72.6 (68.1 , 76.6) | -2.4 |
|  |  |  |  | Wasting | 13 | 15.2 | 15 (11.1 , 19.8) | -0.2 |
|  |  |  |  | Overweight | 0.1 | 0.3 | 0.2 (0.1 , 0.4) | -0.1 |
|  |  |  |  |  |  |  |  |  |
|  | 1997-98 | 6-60 mo | 17471 | Underweight | 47.1 | 38.2 | 42.7 (30.4 , 56) | 4.5 |
|  |  |  |  | Stunting | 54.2 | 61.1 | 59.2 (54 , 64.2) | -1.9 |
|  |  |  |  | Wasting | 6.8 | 7.9 | 8.3 (6.1 , 11.3) | 0.4 |
|  |  |  |  | Overweight | 0.3 | 0.4 | 0.6 (0.4 , 1) | 0.2 |
|  |  |  |  |  |  |  |  |  |
| Nicaragua | 2001 | 0-60 mo | 6468 | Underweight | 9.6 | 7.8 | 8.4 (5.1 , 13.5) | 0.6 |
|  |  |  |  | Stunting | 20.2 | 25.2 | 25.8 (22 , 30.1) | 0.6 |
|  |  |  |  | Wasting | 2 | 2.3 | 2.7 (1.9 , 3.8) | 0.4 |
|  |  |  |  | Overweight | 4.7 | 7.1 | 7.5 (4.7 , 11.8) | 0.4 |
|  |  |  |  |  |  |  |  |  |
| Niger | 1998 | 0-36 mo | 4204 | Underweight | 49.6 | 45 | 45.2 (32.6 , 58.5) | 0.2 |
|  |  |  |  | Stunting | 41.1 | 47 | 47.1 (41.9 , 52.4) | 0.1 |
|  |  |  |  | Wasting | 20.7 | 25.5 | 22.8 (17.4 , 29.3) | -2.7 |
|  |  |  |  | Overweight | 0.8 | 1.2 | 1.5 (0.9 , 2.5) | 0.3 |
|  |  |  |  |  |  |  |  |  |
| Oman | 1994-95 | 0-48 mo | 639 | Underweight | 14.1 | 10 | 12.3 (7.6 , 19.4) | 2.3 |
|  |  |  |  | Stunting | 15.7 | 21.4 | 20.8 (17.5 , 24.5) | -0.6 |
|  |  |  |  | Wasting | 9.1 | 7 | 10.8 (8 , 14.6) | 3.8 |
|  |  |  |  |  |  |  |  |  |
|  | 1998 | 0-60 mo | 14076 | Underweight | 17.8 | 13.1 | 15.6 (9.8 , 24) | 2.5 |
|  |  |  |  | Stunting | 10.4 | 15.9 | 14.5 (12 , 17.3) | -1.4 |
|  |  |  |  | Wasting | 7.2 | 7.8 | 8.7 (6.4 , 11.9) | 0.9 |
|  |  |  |  | Overweight | 1 | 1.7 | 1.9 (1.1 , 3.1) | 0.2 |
|  |  |  |  |  |  |  |  |  |
| Pakistan | 1990-91 | 0-60 mo | 4588 | Underweight | 40.2 | 39 | 36.1 (24.9 , 49.1) | -2.9 |
|  |  |  |  | Stunting | 49.6 | 54.5 | 55 (49.7 , 60.2) | 0.5 |
|  |  |  |  | Wasting | 9.2 | 12.5 | 10.9 (8 , 14.7) | -1.6 |
|  |  |  |  | Overweight | 3.1 | 5.4 | 5.2 (3.2 , 8.3) | -0.2 |
|  |  |  |  |  |  |  |  |  |
| Panama | 1997 | 0-60 mo | 2289 | Underweight | 8.1 | 6.3 | 7.1 (4.3 , 11.5) | 0.8 |
|  |  |  |  | Stunting | 18.2 | 21.5 | 23.6 (20 , 27.7) | 2.1 |
|  |  |  |  | Wasting | 1 | 1.4 | 1.4 (1 , 2) | 0 |
|  |  |  |  | Overweight | 4.2 | 6.2 | 6.8 (4.2 , 10.7) | 0.6 |
|  |  |  |  |  |  |  |  |  |
| Peru | 1991-92 | 0-60 mo | 7205 | Underweight | 10.7 | 8.8 | 9.4 (5.7 , 15) | 0.6 |
|  |  |  |  | Stunting | 31.8 | 37.3 | 38 (33.2 , 43.1) | 0.7 |
|  |  |  |  | Wasting | 1.7 | 1.9 | 2.3 (1.7 , 3.2) | 0.4 |
|  |  |  |  | Overweight | 5.3 | 9.3 | 8.4 (5.2 , 13.1) | -0.9 |
|  |  |  |  |  |  |  |  |  |
|  | 1996 | 0-60 mo | 13829 | Underweight | 7.8 | 5.7 | 6.8 (4.1 , 11.1) | 1.1 |
|  |  |  |  | Stunting | 25.8 | 31.6 | 31.9 (27.4 , 36.6) | 0.3 |
|  |  |  |  | Wasting | 1.1 | 1.6 | 1.6 (1.1 , 2.2) | 0 |
|  |  |  |  | Overweight | 6.4 | 9.9 | 9.9 (6.2 , 15.3) | 0 |
|  |  |  |  |  |  |  |  |  |
| Rwanda | 2000 | 0-60 mo | 6894 | Underweight | 24.3 | 20.3 | 21.4 (13.8 , 31.7) | 1.1 |
|  |  |  |  | Stunting | 42.6 | 48.3 | 48.5 (43.3 , 53.8) | 0.2 |
|  |  |  |  | Wasting | 6.8 | 8.7 | 8.3 (6.1 , 11.3) | -0.4 |
|  |  |  |  | Overweight | 4 | 7.2 | 6.5 (4 , 10.3) | -0.7 |
|  |  |  |  |  |  |  |  |  |
| Sao Tome and Principe | 2000 | 0-60 mo | 1766 | Underweight | 12.9 | 10.1 | 11.3 (6.9 , 17.8) | 1.2 |
|  |  |  |  | Stunting | 28.9 | 35.2 | 35.1 (30.4 , 40) | -0.1 |
|  |  |  |  | Wasting | 3.6 | 3.9 | 4.6 (3.3 , 6.4) | 0.7 |
|  |  |  |  |  |  |  |  |  |
| Sierra Leone | 2000 | 0-60 mo | 2376 | Underweight | 27.2 | 24.7 | 24.1 (15.7 , 35.1) | -0.6 |
|  |  |  |  | Stunting | 33.8 | 38.4 | 40 (35 , 45.2) | 1.6 |
|  |  |  |  | Wasting | 9.9 | 11.6 | 11.7 (8.6 , 15.7) | 0.1 |
|  |  |  |  |  |  |  |  |  |
| Solomon island | 1989 | 0-60 mo | 3980 | Underweight | 21.3 | 16.3 | 18.7 (11.9 , 28.2) | 2.4 |
|  |  |  |  | Stunting | 27.3 | 33.7 | 33.4 (28.9 , 38.3) | -0.3 |
|  |  |  |  | Wasting | 6.6 | 7.4 | 8.1 (5.9 , 11) | 0.7 |
|  |  |  |  | Overweight | 1.1 | 1.9 | 2 (1.2 , 3.3) | 0.1 |
|  |  |  |  |  |  |  |  |  |
| Sri-Lanka | 2000 | 3-60 mo | 2513 | Underweight | 29.4 | 22.8 | 26.1 (17.1 , 37.6) | 3.3 |
|  |  |  |  | Stunting | 13.5 | 18.4 | 18.2 (15.3 , 21.6) | -0.2 |
|  |  |  |  | Wasting | 14 | 15.5 | 16 (11.9 , 21.1) | 0.5 |
|  |  |  |  |  |  |  |  |  |
| Thailand | 1987 | 3-36 mo | 1843 | Underweight | 25.3 | 17.4 | 22.3 (14.4 , 32.9) | 4.9 |
|  |  |  |  | Stunting | 21.5 | 25.3 | 27.3 (23.3 , 31.7) | 2 |
|  |  |  |  | Wasting | 5.3 | 6 | 6.6 (4.8 , 9) | 0.6 |
|  |  |  |  | Overweight | 1.2 | 1.3 | 2.2 (1.4 , 3.6) | 0.9 |
|  |  |  |  |  |  |  |  |  |
| The Former Yugoslav Republic | 1999 | 6-60 mo | 1117 | Underweight | 5.9 | 1.9 | 5.2 (3.1 , 8.5) | 3.3 |
|  |  |  |  | Stunting | 6.9 | 8 | 10.1 (8.3 , 12.2) | 2.1 |
|  |  |  |  | Wasting | 3.6 | 1.7 | 4.6 (3.3 , 6.4) | 2.9 |
|  |  |  |  | Overweight | 4.9 | 9.6 | 7.8 (4.9 , 12.2) | -1.8 |
|  |  |  |  |  |  |  |  |  |
| Togo | 1998 | 0-36 mo | 3603 | Underweight | 25.1 | 23.2 | 22.2 (14.3 , 32.7) | -1 |
|  |  |  |  | Stunting | 21.7 | 29.8 | 27.5 (23.5 , 31.9) | -2.3 |
|  |  |  |  | Wasting | 12.3 | 13.9 | 14.2 (10.6 , 18.9) | 0.3 |
|  |  |  |  | Overweight | 1.5 | 2.6 | 2.7 (1.7 , 4.4) | 0.1 |
|  |  |  |  |  |  |  |  |  |
| Trinidad and Tobago | 2000 | 0-60 mo | 780 | Underweight | 5.9 | 4.4 | 5.2 (3.1 , 8.5) | 0.8 |
|  |  |  |  | Stunting | 3.6 | 5.3 | 5.6 (4.6 , 6.9) | 0.3 |
|  |  |  |  | Wasting | 4.4 | 5.2 | 5.6 (4 , 7.6) | 0.4 |
|  |  |  |  |  |  |  |  |  |
| Turkey | 1998 | 0-60 mo | 2873 | Underweight | 8.3 | 7 | 7.3 (4.4 , 11.8) | 0.3 |
|  |  |  |  | Stunting | 16 | 19.1 | 21.1 (17.8 , 24.9) | 2 |
|  |  |  |  | Wasting | 1.9 | 3 | 2.6 (1.8 , 3.6) | -0.4 |
|  |  |  |  | Overweight | 2.2 | 4 | 3.8 (2.3 , 6.1) | -0.2 |
|  |  |  |  |  |  |  |  |  |
| Zimbabwe | 1999 | 0-60 mo | 3188 | Underweight | 13 | 11.5 | 11.4 (7 , 17.9) | -0.1 |
|  |  |  |  | Stunting | 26.5 | 33.7 | 32.6 (28.1 , 37.4) | -1.1 |
|  |  |  |  | Wasting | 6.4 | 8.5 | 7.8 (5.7 , 10.7) | -0.7 |
|  |  |  |  | Overweight | 7 | 10.6 | 10.7 (6.7 , 16.5) | 0.1 |
